# Case Report: Fading Elk Syndrome in a Herd of Captive Elk (*Cervus elaphus*) in the North American Midwest

**DOI:** 10.3389/fvets.2020.00497

**Published:** 2020-08-21

**Authors:** Paola M. Boggiatto, Lauren S. Crawford, Carly Kanipe, Mitchell V. Palmer, Steven C. Olsen

**Affiliations:** ^1^Infectious Bacterial Diseases Research Unit, National Animal Disease Center, Agricultural Research Service, United States Department of Agriculture, Ames, IA, United States; ^2^Oak Ridge Institute for Science and Education, Oak Ridge, KY, United States; ^3^Immunobiology Graduate Program, Iowa State University, Ames, IA, United States

**Keywords:** case report, fading elk syndrome, elk, ostertagiasis, *Ostertagia*

## Abstract

Fading elk syndrome, or chronic ill-thrift of elk, is a disease associated with abomasal parasitism with *Ostertagia* species, of which elk appear to be particularly susceptible. While this syndrome has been extensively reported to affect wapiti-type red deer hybrids farmed in New Zealand since the mid 1980's, there is only a single report of this disease in North America. Here, we report a case of fading elk syndrome in a herd of 34 elk (*Cervus elaphus*) in Ames, Iowa, at the National Animal Disease Center. Analysis of complete blood counts were unremarkable, but blood chemistry demonstrated a severe hypoalbuminemia. Fecal floatations were also unremarkable, and non-diagnostic. Histological examination of tissues collected at necropsy revealed proliferative abomasitis and nematodes consistent with *Ostertagia* spp. Anthelmintic treatment consisting of a combination of pour-on Cydectin^®^ and injectable Noromectin Plus^®^, at double the recommended dose for cattle, showed positive results, as all remaining animals in the herd recovered. The work presented here is the first report of naturally-acquired disease in a herd of captive elk used for research and sheds light on this seldomly-reported disease in North America.

## Introduction

Fading elk syndrome, also known as chronic ill-thrift, is a disease characterized by progressive weight loss and sometimes severe cachexia. This often fatal disease has been reported in wapiti or elk (*Cervus elaphus*) and in wapiti-type red deer hybrids farmed in New Zealand since the mid 1980's (1). However, a search of the literature showed only a single report of fading elk syndrome in North America. The study described this syndrome in a large herd of farmed elk in Maine, with 84% mortality rate despite supportive nutritional and medical intervention unmistakably demonstrating the severe effects of this disease (2).

Typically, fading elk syndrome affects animals >1 year old. Clinical signs may be present for months as anorectic animals progressively lose body condition and become emaciated. The presence of diarrhea is a more variable clinical sign. Clinical pathologic features include hypoalbuminemia, hypocuprosis, elevated abomasal pH, and elevated plasma pepsinogen levels (3). Affected animals are often refractory to treatment and succumb to the disease.

Prior to the 1990's fading elk syndrome was linked to a variety of factors including environmental stresses, poor nutrition, copper and vitamin deficiencies, infectious agents, genetics, and parasitism (4). As such, treatment regimens included various combinations of vitamins, antibiotics, anthelmintics, probiotics, and steroids (1, 5). In the early 1990's, a link between this syndrome and gastrointestinal nematodiasis, particularly abomasal parasitism with *Ostertagia* spp. was shown (6). Nevertheless, despite the identification of an etiologic agent, treatment is not always successful (1, 5–7).

As with other ruminants, elk are susceptible to infection with a wide range of nematodes. Several genera have been reported in North American elk, including five *Ostertagia* species (8). Adult *Ostertagia* reside in the abomasal lumen but larvae develop within the gastric glands resulting in loss of both parietal and mucous cells. Abomasal mucosal damage leads to severe metabolic consequences, protein loss, and energy spent both in tissue repair and in mounting an immune response to the infection [reviewed in (9)]. Diagnosis of ostertagiasis in elk is difficult given the insidious and protracted nature of the disease, the unreliability of fecal egg counts, and the fact that the heavy winter coat of elk can easily hide poor body condition.

Nematode infections in domestic ruminants cause major production losses worldwide. Production loss in farmed elk can be equally detrimental, yet there are very few reports of clinical disease associated with nematode infection in elk. In the present study, we a case of fading elk syndrome in a captive elk herd in the North American Midwest. The aim of this report is to describe the clinical presentation, clinical pathology, histopathologic changes, therapeutic intervention, and response to treatment in surviving animals. To our knowledge, this is the first report of this naturally-acquired disease in a captive elk herd used for research purposes.

## Case Description

In July 2019, six 2-year old elk (*Cervus elaphus*) hinds were noted to be dull and depressed. The six animals were part of a herd of 17 animals (16 hinds and 1 bull), located on the National Animal Disease Center (NADC) campus in Ames, Iowa. Collectively, the herd was also noted to be anorexic for a few days prior to the identification of clinical signs. Closer examination revealed the six depressed hinds to be in poor body condition and had failed to completely shed their winter coats.

A second herd, consisting of 17 animals (16 hinds and 1 bull), housed in an adjacent pasture was also evaluated. Only one hind (#84) in this group was found to be in poor body condition. Blood samples from clinical animals were obtained for complete blood counts, serum chemistries, and infectious diagnostic testing. Fecal samples were also collected.

Complete blood counts (CBC) were unremarkable for all animals tested. However, serum chemistries (Table 1) showed a marked hypoalbuminemia. In two of the animals (#100 and #113), serum albumin levels were below the instrument's level of detection (i.e., < 1 mg/ml). Fecal floatation examinations were performed on individual animals, as well as from pooled fecal samples. Findings revealed occasional strongyle-type eggs (1–10 per slide) and occasional operculated eggs consistent with a trematode spp.

**Table 1:** Serum chemistry results for clinical female elk at the time of clinical presentation.

		**Animal number**						
	**Reference range**	**84**	**100**	**101**	**102**	**104**	**110**	**113**
Albumin	2.9–3.9 g/dL	**1.5**	** <1.0**	**1.5**	**1.1**	**1.1**	**1.7**	** <1.0**
Total bilirubin	0.1–0.7 mg/dL	0.4	0.4	0.3	0.4	0.4	0.4	0.3
BUN	11–33 mg/dL	24	31	26	**35**	31	29	30
Calcium	8.5–10.2 mg/dL	8.8	9.4	8	9.9	8.7	9.2	9.2
Phosphorous	2.6–7.5 mg/dL	6.2	4.6	7.4	5.6	5.5	5.9	2.8
Creatinine	1.59–2.72 mg/dL	2	1.9	1.8	2.5	2.3	**1.5**	2
Glucose	109–288 mg/dL	100	127	127	135	169	119	206
Sodium	133–154 mmol/L	139	142	140	142	140	141	139
Potassium	4.1–15.7 mmol/L	4.7	7.3	5.7	5.9	6.7	5.2	>8.5
Total protein	5.4–8.1 g/dL	**5.0**	**4.4**	5.8	5.5	**5.3**	5.8	**5.2**
Globulins	2.7–4.9 g%	3.5	NA	4.3	4.3	4.2	4.1	NA

*Highlighted in bold are out of range values*.

Blood and serum samples from all affected animals were submitted to the National Veterinary Service Laboratories (Ames, IA) for bluetongue virus (BTV), epizootic hemorrhagic disease virus (EHDV), malignant catarrhal fever (MCF), and bovine leukemia virus (BLV) testing. Three of the clinical animals were seropositive for EHDV, but no virus was recovered via PCR from any of the tissues submitted, suggesting exposure to EHDV, but no ongoing infection. All other test results were negative. Fecal samples were negative for Johne's (*Mycobacterium avium* subsp. *paratuberculosis*) via culture and polymerase chain reaction (PCR) for the IS900 genetic element.

**Figure 1:**
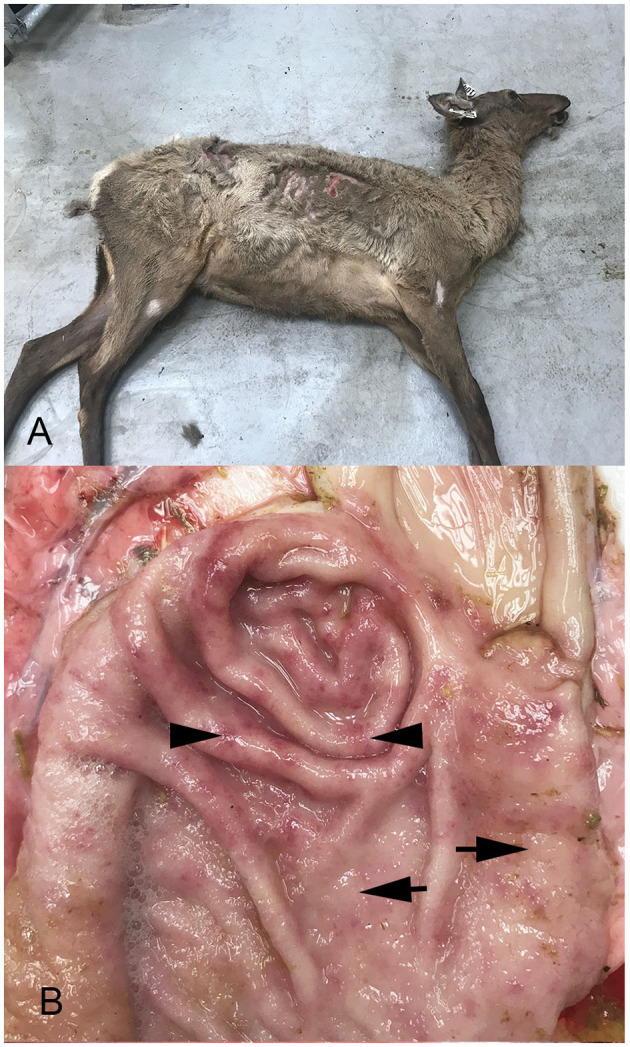
Photographs of an elk hind showing poor body condition, and unshed winter coat **(A)** and abomasum from one animal **(B)**. Note increased mucus, mucosal thickening characterized by raised, pale, nodular foci (arrows), and coalesced areas of hyperemia (arrow heads).

At necropsy, all four elk were thin, with rough hair coats (Figure 1A) and decreased subcutaneous and renal fat stores. One of three hinds had fecal staining of the perineum consistent with diarrhea, while the remaining hinds had normal formed feces.

Abomasa from three of the four hinds were characterized by variable degrees of irregular abomasal mucosal thickening, which was most prominent in the fundus, but also extended well into the body. In one animal, the abomasal mucosa was cobblestone in appearance, with numerous raised, pale, nodular foci, 5–10 mm in diameter (Figure 1B), that coalesced in severely affected areas. In all four animals, the mucosal surface was covered with moderate to large amounts of mucus, while scattered throughout the thickened abomasal mucosa were small 1–3 mm red foci. At all levels of the small intestine, the mucosa was diffusely hyperemic. The cecum was less affected, with small multiple hyperemic foci. More distal regions of the large intestine were normal in appearance.

**Figure 2:**
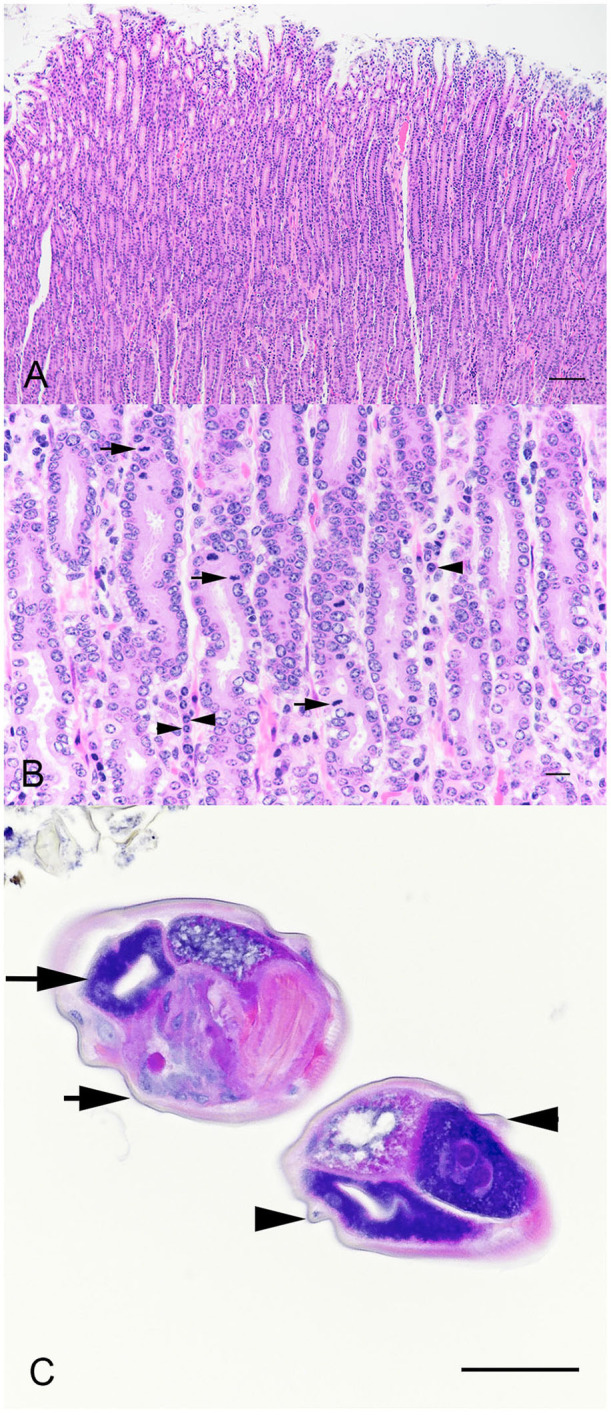
Photomicrographs of sections of thickened abom asal mucosa demonstrating elongated gastric pits **(A)**. Bar = 200 μm. Tightly packed mucous cells with increased mitotic figures (**B**, arrows), and plasma cells (arrow heads). Bar = 25 μm. Cross sections of a nematode within the abomasal lumen **(C)**. Morphologic features were typical of *Trichostrongyles*, including a thin cuticle (arrow heads) with prominent longitudinal ridges (arrow heads) and a platymyarian-meromyarian musculature (arrows). Bar = 75 μm.

Sections of abomasum from all four hinds were characterized by variable degrees of hyperplasia and metaplasia. Elongated gastric pits were lined by tightly packed mucous cells with increased mitotic figures (mucous cell hyperplasia) resulting in moderate thickening of the abomasal mucosal layer (Figure 2A). Multifocally, mucous cells replace gastric parietal cells (mucous cell metaplasia). Within the lamina propria were moderately increased numbers of lymphocytes, plasma cells, and eosinophils (Figure 2B). Occasional dense aggregates of lymphocytes (lymphoid nodules) were seen in the abomasal lamina propria. Within the abomasal lumen multiple cross sections of a nematode, roughly 100 μm in diameter, were present (Figure 2C). Morphologic features were typical of *Trichostrongyles*, including a thin cuticle with prominent longitudinal ridges, pseudocoelom, and a platymyarian-meromyarian musculature (Figure 2C). The digestive tract was typical oligocytous strongylin with few visible cells and multiple nuclei.

### Diagnosis and Therapeutic Intervention

Based on our initial clinical findings of poor body condition, diarrhea, and severe hypoalbuminemia, with no clinical pathologic evidence of hepatic or renal involvement, we proposed a tentative diagnosis of protein-losing enteropathy. Gross and histological findings of proliferative abomasitis and identification of nematodes consistent with *Ostertagia* spp. provided a definitive diagnosis of protein-losing enteropathy due to ostertagiasis. Furthermore, identification of increased number of lymphocytes, eosinophils, and plasma cells in the abomasum, confirmed type II ostertagiasis.

Anti-helminthic treatment was started immediately and consisted of a combination of transdermal Cydectin^®^ (5 mg moxidectin/ml, 5 ml/kg; Bayer Healthcare, Shawnee Mission, KS) and injectable Noromectin^®^ Plus (1% ivermectin and 10% clorsulon; 2 ml/45 kg; Norbrook Inc, Overland Park, KS). Clinical animals also received vitamin B complex (1 ml/45 kg; Huvepharma, Inc., St. Joseph, MO), administered subcutaneously. Five weeks after presentation of clinical signs, all elk were reevaluated. Previously-clinical animals remained thin, but their body condition had started to improve. Blood samples were collected and serum chemistries reanalyzed 5 weeks following treatment. In three of the four animals, albumin levels had started to improve (Table 2). Ten weeks after initial treatment, all animals were re-treated with injectable Noromectin^®^ Plus, and serum chemistries were again evaluated in previously-clinical animals (Table 2). At this sampling timepoint, serum albumin levels were within normal range for all animals analyzed.

**Table 2:** Serum chemistry results 5 and 10 weeks following the initiation of clinical signs.

		**5 weeks**				**10 weeks**		
		**Animal number**						
	**Reference range**	**84**	**101**	**102**	**110**	**101**	**102**	**110**
Albumin	2.9–3.9 g/dL	**1.8**	2.9	**2.7**	**2.7**	3.6	3.6	3.4
Total bilirubin	0.1–0.7 mg/dL	0.3	0.2	0.3	0.3	0.3	0.3	0.2
BUN	11–33 mg/dL	30	32	**36**	32	30	33	**35**
Calcium	8.5–10.2 mg/dL	9.4	10.1	9.9	9.8	9.4	**10.3**	9.4
Phosphorous	2.6–7.5 mg/dL	6.8	8.7	8.2	7.4	**8.2**	7.5	**9.3**
Creatinine	1.59–2.72 mg/dL	1.6	1.3	1.7	1.3	**1.2**	1.6	**1.3**
Glucose	109–288 mg/dL	**101**	141	116	**102**	164	119	120
Sodium	133–154 mmol/L	143	141	139	141	142	145	139
Potassium	4.1–15.7 mmol/L	5.1	5.2	5.2	4.6	5.8	5.5	4.8
Total protein	5.4–8.1 g/dL	**5.3**	7.6	7.2	7.6	7.5	7.5	7.4
Globulins	2.7–4.9 g%	3.5	4.7	4.5	4.9	3.9	3.9	4.1

*Highlighted in bold are out of range values*.

## Discussion

Fading elk syndrome, also known as chronic ill-thrift of elk is characterized by progressive weight loss, severe cachexia, and it is often fatal. Abomasal parasitism with *Ostertagia* spp. has been shown to be associated fading elk syndrome (10). Reports of this disease in other parts of the world are common, yet only a single report of fading elk syndrome in North America was found in the literature (2).

Ostertagiines, are nematodes belonging to the family Trichostrongylidae, and are the most pathogenic strongyles in ruminants (11). In North America, five species of *Ostertagia* have been identified as causing disease in elk and include *O. leptospicularis/O. kolchida, O. ostertagi/O. lyrata, O. gruehneri/O. arctica*, and *O. mossi/O. dikmansi* (12–14). Distinction between species can be made based on morphological characteristics of adult males. As with other ostertagiines, *Ostertagia* spp. have a direct life cycle consisting of two stages, the free-living and parasitic stages. In the host, adults reside in the abomasum and mature females release embryonated eggs that are shed in the feces. Once in the environment, eggs hatch, and the larvae (L) undergo several development stages, L1-3. Infective L3 are ingested during grazing and pass to the abomasum where they penetrate gastric glands. Further molting within the gastric gland results in L4 and L5 stages. Young adult worms emerge from the gastric glands and continue their maturation to fully mature adults on the mucosal surface of the abomasum. While the typical pre-patent period for *Ostertagia* spp. is of ~21 days, L3 larvae can arrest their development the gastric glands as inhibited L4, a phenomenon termed hypobiosis, which can last up to 7 months. The pathology of clinical ostertagiasis is mediated by a large number of L3 larvae developing into adults over a short period of time, which results in damage to the abomasal mucosa. Clinical presentation can range from mild (reduced growth) to severe (rapid weight loss, diarrhea, anemia, and death). Type II ostertagiasis occurs in older animals and is mediated by the emergence of immature adults from gastric glands as hypobiotic larvae resume their development. This can take place weeks to months following initial ingestion of L3. Severity of disease is dependent on the emergence of parasites. If this emergence is gradual, clinical signs are protracted. However, if emergence occurs rapidly, and in large numbers, clinical signs are rapid and severe (15).

The pathophysiology of ostertagiasis is centered around the damage to the gastric glands in the abomasum. Physical damage compromises intracellular junctions, leading to movement of serum proteins, particularly albumin, into the abomasal lumen. Increased levels of albumin in the gastrointestinal lumen decreases fluid absorption, resulting in diarrhea, and the loss of serum albumin can lead to third spacing and/or ascites. The destruction of the parietal cells results in increased abomasal pH (15). Elevated abomasal pH compromises protein digestion, both through an inability to denature proteins and decreased conversion of pepsinogen into its active form, pepsin. Increased abomasal pH also decreases absorption of minerals such as copper, and triggers the production of gastrin, which has been correlated to inappetence (16). Depression of host appetite is one of the most significant systemic changes and is considered a major contributing factor to impaired nutrition (9). Increased pH can also alter normal abomasal flora, contributing to the development of diarrhea. Altogether, the inability to efficiently metabolize protein, the concurrent protein-losing enteropathy, and the decreased feed intake contribute to weight loss and general ill-thrift of affected animals.

Diagnosis of type II ostertagiasis is more complicated given the increased complexity in its life cycle (i.e., hypobiotic stage), variations in emergence due to climatic conditions, and gradations or severity of clinical disease (15). Important variables that may aid in the ante-mortem diagnosis of type II disease include herd history, herd density, anti-helminthic use practices, pasture management, age of animals affected, and time of year. Clinical presentation characterized by weight loss, rough coats, and diarrhea is consistent with intestinal parasitism, but certainly not specific, as these clinical signs may result from various other diseases. Fecal egg counts should be used with caution, as they may not be useful in reaching a diagnosis, but they can be a good indicator of high adult worm burden (2, 8). Aside from histological identification of parasite cross-sections, we were unable to isolate adult worms from the abomasal mucosa. While we were unable to identify the *Ostertagia* species responsible for disease in this herd, variations in histopathologic changes and clinical presentation among species of *Ostertagia* have not been reported. Gross and histological changes observed post-mortem were consistent with type II ostertagiosis. Gross lesions are characterized by multiple pale, raised nodules on the mucosal surface, accompanied by mucosal reddening and edema (15). The classic “Morrocan leather” or “cobblestone” appearance, occurs in severe cases as lesions coalesce. Despite the severe clinical disease observed in the hinds in this study, gross lesions were distinguishable in three of the affected hinds, but only one showed the classic “cobblestone” pattern.

Typically, affected animals are refractory to therapeutic intervention and in North America, there are no anthelmintic approved for use in deer or elk. However, several anthelmintic have been tried including macrocyclic lactones and benzimidazoles. Oral anthelmintic, at dosages for cattle, have poor results in both deer and elk (5, 17). Double the cattle dose can clear the adult forms but only has ~40% efficiency against encysted larvae (6). In general, oral anthelmintic administration should be avoided, as the elevated pH of the abomasum interferes with absorption (6, 7). Instead, parenteral or pour-on administration is recommended. Pour-on ivermectin at triple the recommended dose can significantly reduce adult worm burden and prevents albumin loss, but it does not significantly lower larval forms (18). Additionally, there are anectodal reports of skin irritation and anorexia observed in some animals within a day or two following treatment when pour-on ivermectin is used on animals with summer coats (8). Moxidectin pour-on was shown to be highly effective against mature and immature abomasal nematodes in wapiti hybrid deer and red deer (19). Additionally, moxidectin was shown to be highly effective at preventing reinfection in red deer for up to 42 days post-treatment (20). However, it should be noted that resistance due to overuse of moxidectin pour-on has been documented in New Zealand (21), so as with other anthelmintic, judicious use should be practiced. A moxidectin injectable and oral oxfendazole/levamisole combination has also been reported, and was shown to achieve a 98% and 97.5% efficacy against larval and adult stages (4).

In this case report, we utilized a combination of pour-on Cydectin^®^ and injectable Noromectin Plus^®^ at double the recommended dose for cattle. This particular treatment regimen showed positive results, as all remaining animals in the herd recovered, and albumin levels had improved within 5 weeks of starting treatment, and were within normal limits by 10 weeks after the identification of clinical signs. Additionally, we did not observe any adverse effects from treatment at the administered doses, such as anorexia, injection site reaction, or alopecia.

Our facility has housed farmed elk for the last 20 years, yet this was the first encounter with intestinal parasites resulting in clinical disease. It is possible that this group of elk acquired the infection prior to arrival at our facility. However, given the timing of clinical signs, it is more likely that the animals acquired the infection once on campus. The pastures have been used to house elk for several years, and prior anthelmintic treatment may not have been sufficient to control shedding, resulting in pastures with high larval burden. However, we were unable to determine the exact origin of the infection.

The work presented here sheds light on fading elk syndrome, a seldom reported disease of elk in North America. This highly debilitating, and often fatal, condition can have a detrimental effect on captive elk herds, in both in terms of production and animal loss. Elk appear to be more susceptible to abomasal nematodes than red deer, and don't appear to develop immunity with age. This phenomenon may be related to the fact that elk have not evolved with these parasites, and therefore, are more susceptible (6). Diagnosis of fading elk syndrome can be difficult, and therapeutic intervention is not always successful. Therefore, control measures, including good husbandry, accurate herd history, pasture management, and regular anthelmintic treatment are the best approach for dealing with this disease. Since animals may remain subclinical for extended periods of time and fecal egg counts are not good indicators of disease, routine, and proper parasite control annually or biannually of the whole herd should be practiced.

## Data Availability Statement

All datasets generated for this study are included in the article/supplementary material.

## Ethics Statement

Written informed consent from the owner for the publication of these animals' cases was not required as the animals concerned were the property of the USDA.

## Author Contributions

PB, LC, and SO performed all veterinary work including clinical assessment, sample collection, treatment administration, and follow-up. CK and MP performed necropsies and histopathology. PB wrote the manuscript. LC, CK, MP, and SO edited the manuscript. All authors contributed to the article and approved the submitted version.

## Conflict of Interest

The authors declare that the research was conducted in the absence of any commercial or financial relationships that could be construed as a potential conflict of interest.
